# Beyond Birth: Pioneering Insights into Colostrum Quality Variation among Bitches with Different Types of Parturition

**DOI:** 10.3390/vetsci11030114

**Published:** 2024-03-03

**Authors:** Maja Zakošek Pipan, Meta Sterniša, Tanja Plavec

**Affiliations:** 1 Clinic for Reproduction and Large Animals, Veterinary Faculty, University of Ljubljana, Gerbičeva 60, 1000 Ljubljana, Slovenia; maja.zakosekpipan@vf.uni-lj.si; 2 Biotechnical Faculty, University of Ljubljana, Jamnikarjeva 101, 1000 Ljubljana, Slovenia; meta.sternisa@bf.uni-lj.si; 3 Small Animal Clinic, Veterinary Faculty, University of Ljubljana, Gerbičeva 60, 1000 Ljubljana, Slovenia

**Keywords:** canine, dogs, neonate, colostrum, IgG, IgA, aglepristone

## Abstract

**Simple Summary:**

In this study, the immunological quality of the colostrum of dogs was examined in relation to the different modes of birth in bitches. The four following groups were studied: vaginal delivery (VP), emergency cesarean section (EM-CS), elective cesarean section, (EL-CS) and elective cesarean section with a prior aglepristone injection (EL-A). Colostrum samples were taken from 40 bitches of 18 breeds immediately after the birth of the first puppy or directly after surgery, and four hours later. The concentrations of immunoglobulin G (IgG) and IgA were measured using enzyme-linked immunosorbent assay (ELISA) tests. The EL-CS group showed significantly lower IgG and IgA concentrations when compared to the other groups. The injection of aglepristone 24 h before the cesarean section increased the IgG and IgA concentrations. Despite lower IgG and IgA levels in the EL-CS group, puppy survival was not affected. The study suggests that progesterone withdrawal before birth may influence higher immunoglobulin concentrations in the mammary glands of bitches.

**Abstract:**

This study deals with the immunological quality of canine colostrum in relation to the different parturition modes in bitches. It included four groups of bitches, who underwent vaginal parturition (VP), emergency cesarean section (EM-CS), elective cesarean section (EL-CS), or elective cesarean section with aglepristone injection 24 h prior to surgery (EL-A). Colostrum samples from 40 bitches of 18 breeds were taken immediately after the birth of the first puppy or directly after surgery, and four hours later. The concentrations of immunoglobulin G (IgG) and IgA were measured using ELISA tests. The initial IgG concentration was 18.3 ± 10.2 g/L, and the IgA concentration was 13.7 ± 5.8 g/L, respectively. Significantly lower IgG and IgA concentrations were observed in the EL-CS group compared to other groups. The administration of aglepristone led to an increase in IgG and IgA concentrations. Despite the lower immunoglobulin levels with EL-CS, the survival rate of the puppies was not affected. The study shows that immunoglobulin concentrations in colostrum vary between bitches, with the type of birth significantly influencing the levels. Progesterone withdrawal before birth could play a decisive role in increasing IgG and IgA concentrations in the mammary glands of the bitches.

## 1. Introduction

Colostrum, affectionately known as the “first mother’s milk”, is a valued and nutrient-rich substance produced by female mammals, including dogs, in the first days after birth. It is important in the first stages of newborn puppies, and is characterized by a unique composition enriched with essential components, such as proteins, fats, vitamins, minerals, and antibodies [1,2]. One of the most important properties of colostrum is its content of antibodies, which act as guardians of passive immunity and form a protective shield for newborn puppies, protecting them from various infections and diseases until their own immune system matures. This is of the utmost importance, as neonatal mortality in this species is a significant risk, with morbidity rates ranging from 17% to 26%. Most of these losses, approximately 70–90%, occur within the first week of life and are predominantly due to infectious diseases [3,4].

Unlike some other species, the canine endoteliochorial placenta has a limited ability to transfer immunoglobulins, underscoring the central role of colostrum in the nurturing of puppies during the initial three weeks of their lives. Colostrum is a transient supply produced exclusively in the mammary glands in the initial days following birth, heralding the transition to regular milk production. Therefore, it is essential that newborn puppies receive it immediately following birth [4,5].

In domesticated animals, the concentration of immunoglobulins in colostrum plays a pivotal role in facilitating the successful transmission of passive immunity to the newborn offspring. This concentration exhibits significant variability among females, with the levels of IgG ranging from 11.7 to 101.4 g/L in sows [6,7], and from 25.7 to 168.7 g/L in cows [8,9]. In dogs, the immune quality of colostrum varies widely from bitch to bitch, and even between different mammary glands of the same bitch. Studies show a mean IgG concentration in colostrum of about 20.8 ± 8.1 g/L [10]. Nevertheless, research on the immune quality of canine colostrum is still relatively unexplored, with few studies providing IgG concentration values from specific breeds [10,11].

Birth is a highly organized event with complex and sometimes puzzling hormonal signals. While in certain animal species, such as pigs, cows, goats, mice, cats, and rabbits, cortisol-induced and placenta-derived prostaglandin F2α (PGF2α) plays a critical role in driving luteolysis [12], the scenario differs significantly in humans, other primates, and guinea pigs [13]. In these species, birth is characterized by an increased circulating progesterone level with a simultaneous reduction in placental progesterone signaling [14].

However, the dog has a unique endocrinological history among all domestic mammals. Unlike other species, dogs do not exhibit steroidogenic activity in the placenta. Instead, progesterone is produced exclusively by the corpus luteum (CL), resulting in no birth-specific increase in estrogen levels. In addition, antiluteolytic mechanisms are absent in the early canine diestrus phase, resulting in naturally regulated and prolonged CL activity [14].

In the endotheliochorial placenta of dogs, decidual cells derived from the maternal stroma exclusively express nuclear progesterone receptors (PGRs). The importance of this distribution of PGR is particularly evident in the birth process [15]. The decrease in circulating progesterone levels before parturition or the functional inhibition of PGR via antigestagen agents such as aglepristone results in decreased progesterone/PGR signaling by the decidual cells. This, in turn, initiates an era of increased luteolytic PGF2α production by the trophoblast which eventually leads to parturition or, in some cases, abortion [16].

Aglepristone (RU 46534), a competitive progesterone antagonist used to treat various progesterone-dependent physiologic or pathologic conditions, shows a remarkable ability to induce labor in 100% of female dogs when administered immediately before parturition [17,18,19]. However, planning an elective cesarean section raises concerns about the potential risk of premature birth. A study conducted by Levy et al. [20] highlights the promising prospects of aglepristone in reducing these risks. Administering aglepristone within a span of 1 to 2 days prior to the expected date of delivery, provided that ovulation is accurately determined at the time of mating, proves to be a strategic measure to address these concerns. In view of this fact, we were interested in exploring the influence of various delivery methods, such as natural birth, planned cesarean section, and cesarean section following aglepristone administration, on the IgG and IgA levels in colostrum. This investigation constitutes the primary focus of our study.

## 2. Materials and Methods

### 2.1. Animals

The study was approved by the Faculty Ethics Committee. The owners who volunteered their dogs for this study signed a consent form for the collection of samples from the bitches. They were recruited between October 2019 and May 2023, and received only Food and Drug Administration (FDA)-approved food for pregnant dams. Forty bitches participated in the study, and the litter sizes ranged from two to ten puppies per litter. The types of deliveries were divided into vaginal delivery (VP, n = 13), emergency cesarean section (EM-CS, n = 8), elective cesarean section (EL-CS, n = 11), and elective cesarean section with an aglepristone (Alizin, Virbac, Medical-Intertrade, Slovenia) 10 mg/kg injection 18–24 h before the procedure (EL-A, n = 8). Each newborn puppy was assessed using the modified Apgar scoring system [21], examined for externally visible congenital malformations and then weighed. Resuscitation and supportive measures were performed as indicated.

### 2.2. Cesarean Section (CS)

Emergency CS was performed when the medical management of dystocia had failed or was inadvisable, according to the indications described previously [22,23]. Elective CS was scheduled 63 days after ovulation. This determination was further supported with the repeated evaluation of blood progesterone concentrations and fetal ultrasonographic measurements, as reported by Meloni in 2015 [24]. Prior to the surgery, the health condition of the female dog was assessed via clinical examination and a complete blood count. During the preoxygenation, the abdominal area was aseptically prepared. The cesarean section procedure consistently adhered to the same anesthesia protocol. After induction with propofol (Fresenius Kabi Ltd., Runcorn, Great Britain) at a dose of 4–7 mg/kg intravenously (IV), the dogs were intubated and general anesthesia was maintained using 1.5–3% sevoflurane (Sevoflurane, Dräger, Lübeck, Germany). Analgesia with methadone (Comfortan, Dechra, Northwich, Great Britain) 0.2 mg/kg subcutaneously (SC) was administered after the first incision. During the procedure, a crystalloid solution (Hartmann solution, B. Braun, Melsungen, Germany) 5–10 mL/kg/h IV was administered. The cesarean section was conducted with a caudal celiotomy, followed by a ventral hysterotomy. Fetal removal commenced with the caudalmost fetus, proceeding cranially. After closing the hysterotomy site, oxytocin (Oxytocin, veyx-Pharma Gmbh, Schwarzenborn, Germany) 0.25–1 international unit (IU) was injected into the uterine wall. The abdominal cavity was rinsed with warm 0.9% sodium chloride (NaCl, B. Braun, Melsungen, Germany) and closed in a routine manner. Four hours after the surgical incision, methadone 0.2 mg/kg SC was administered. Oral tramadol chloride (Tramal, Stada, Bad Vilbel, Germany) at 3 mg/kg was administered every 12 h for up to three days postoperatively. The bitches and their puppies were discharged from the clinic when the bitches displayed typical maternal behavior.

### 2.3. Vaginal Parturition

Mothers were clinically examined at the time of delivery and 5–7 days earlier to ascertain their normal clinical status, and VP took place in their home environment. 

### 2.4. Colostrum Collection

Before collecting the samples from the caudal pair of nipples, they were disinfected using a solution of propan-2-ol and benzalkonium chloride, and the collection of samples was performed while wearing sterile gloves. The colostrum was frozen at −80 °C within 60 min and stored for 6 months until analysis. In the VP group, colostrum samples were obtained right after the first puppy’s birth and four hours later. In the CS bitches, the samples were collected immediately after the CS and once more four hours after the surgery. Following the sampling process, the nipples were wiped with a sterile Hartmann’s solution to ensure cleanliness before allowing the puppies to nurse.

### 2.5. IgA and IgG Assay

The concentration of IgG and IgA in colostrum was assessed using a commercial ELISA test, following the manufacturer’s guidelines (IgG A and IgG ELISA Kit, Abcam, Cambridge, UK). Colostrum samples were initially thawed at room temperature, and then centrifuged (30 min, 2000× *g*, 4 °C) prior to IgG and IgA evaluation [10]. Fat-free whey underwent dilution at ratios of 1:100,000 and 1:400,000 for IgG, and 1:4000 and 1:8000 for IgA, correspondingly. Within one plate, the repeatability of the colostrum assay, as indicated by the intra-assay coefficient of variation, stood at 3.7% for IgG and 2.9% for IgA. 

### 2.6. Statistical Analysis

The findings are presented as mean ± standard deviation. The linear mixed model (breed size as fixed and individual bitch as random effect) showed that breed size had no influence on IgG (*p* = 0.1449) and IgA (*p* = 0.455) concentrations. Thus, the results of IgG and IgA concentrations were analyzed using mixed ANOVA, followed by pairwise comparisons between groups using the estimated marginal means with Bonferroni adjustment. Apgar scores were compared with nonparametric Kruskal–Wallis rank sum tests, followed by pairwise comparisons using Wilcoxon rank sum test. *p* < 0.05 was considered significant. The statistical analysis was conducted utilizing R 4.3.2 software (R Core Team, 2023).

## 3. Results

### 3.1. Basic Information

During the study, 204 puppies were born to 40 healthy bitches; among them, 18 different breeds were represented. Thirteen stillborn puppies were not included in the study. Data on the number of bitches and puppies are presented in Table 1. The mothers were between 22 and 90 months old (mean 41.1 ± 15.1 months). There were 13 VPs, 11 EL-CSs, 8 EM-CSs, and 8 CS-As. The mean litter size was 5.10 ± 2.17 puppies. Table 2 shows the number and proportion (%) of puppies by the type of parturition, sex, and survival, and the number of puppies in each size group by type of birth is shown in Table 3. 

Three puppies from a single bitch died within the first 48 h. According to the owner’s observations, the bitch showed inadequate maternal instincts and displayed aggression towards her offspring. These puppies were part of a large VP litter of 12 puppies who were born to a Great Dane.

Within the first week of life, nine puppies from six different females (1 VP, 2 EL-CS, and 3 EM-CS) died. Post-mortem examinations revealed interstitial pneumonia in seven puppies, but no infectious agent was identified. Two other puppies had bloating, a distended abdomen, and signs of flatulence. Despite rigorous treatment, the local veterinarian was unable to save the puppies.

At three weeks of age, four puppies from four different females (2 VP, 1 EL-CS, and 1 EL-A) died without an autopsy being performed, so the cause of death remained unclear. 

### 3.2. IgG and IgA Concentrations

**Figure 1:**
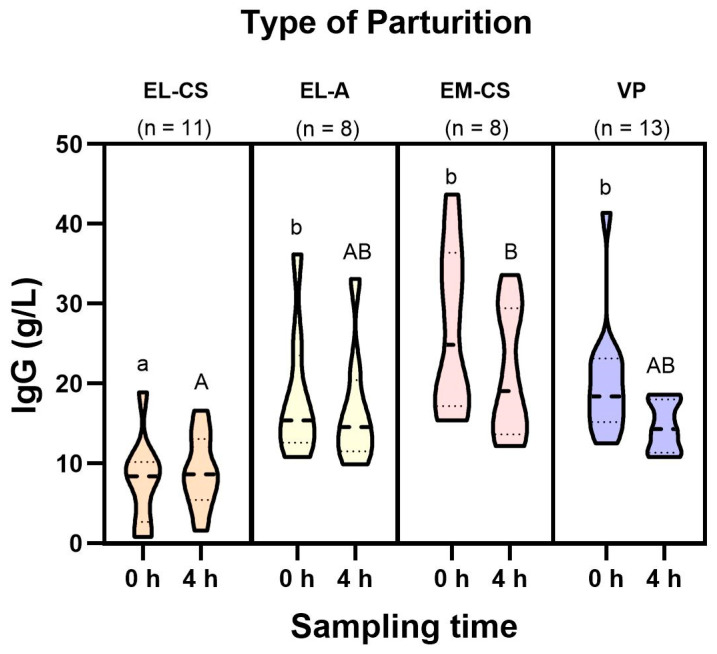
IgG concentration (g/L) in colostrum was determined 0 and 4 h after birth. Different small and capital letters above the violin plots indicate significant differences (*p* < 0.001) between the IgG concentrations, according to the type of parturition at 0 h and 4 h, respectively.

The IgG concentration in colostrum at birth ranged from 0.8–43.7 g/L (mean: 18.3 ± 10.2 g/L) to 1.6–33.6 g/L (mean: 15.0 ± 7.3 g/L) four hours after birth. As shown in Figure 1, the IgG concentration in the EL-CS group was lower than the concentrations in the other three groups. The mixed ANOVA showed differences for the effect of interaction time × type of parturition (F(3,30) = 9.282, *p* = 0.022). Bonferroni-corrected pairwise comparisons revealed differences between EL-CS and EL-A (−3.03, *p* < 0.001), EL-CS and EM-CS (−5.48, *p* < 0.001), EL-CS and VP (−3.97, *p* < 0.001) at time 0 h, and between EL-CS and EM-CS (−3.68, *p* < 0.001) at time 4 h. When aglepristone was administered 24 h before labor induction (EL-A), the IgG levels increased comparatively to the group without aglepristone application (EL-CS), which was statistically significant immediately after birth (*p* < 0.001). The IgG concentrations in the EL-A group were comparable to the concentrations achieved in the EM-CS and VP groups. There were no statistically significant differences in IgG concentrations between the sampling times within each group.

**Figure 2:**
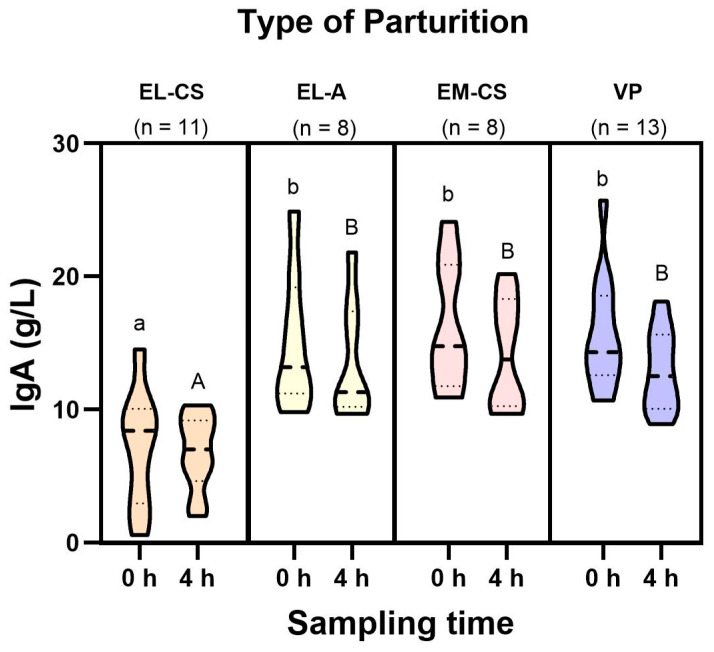
IgA concentration (g/L) in colostrum was determined 0 and 4 h after weaning. Different small and capital letters above the violin plots indicate significant differences (*p* < 0.05) between IgA concentrations according to type of parturition at 0 h and 4 h, respectively.

The IgA concentration in colostrum at birth varied between 0.6–25.7 g/L (mean: 13.7 g/L +/− 5.8) and 2.0–21.8 g/L (mean: 11.7 g/L ± 4.6) four hours after birth. As shown in Figure 2, the pattern of concentrations achieved was similar to IgG. The IgA concentration in the EL-CS group was lower than the concentrations achieved in the other three groups at both time points. The mixed ANOVA showed differences for the effect of interaction time × type of parturition (F(3,30) = 1.267, *p* = 0.003). Bonferroni-corrected pairwise comparisons revealed differences between EL-CS and EL-A (−3.88, *p* < 0.001), EL-CS and EM-CS (−4.52, *p* < 0.001), EL-CS and VP (−4.72, *p* < 0.001) at time 0 h, and between EL-CS and EL-A (−3.42, *p* < 0.001), EL-CS and EM-CS (−3.75, *p* < 0.001), and EL-CS and VP (−3.27, *p* < 0.001) at a time of 4 h. When aglepristone was administered 24 h before labor induction (EL-A), IgA concentrations increased comparatively to the group without aglepristone application (EL-CS) (*p* < 0.001). The IgA concentrations in the EL-A group were comparable to the concentrations achieved in the EM-CS and VP groups. There were no statistically significant differences in IgA concentrations between sampling times within each group.

### 3.3. Assessment of Apgar Score within 5 Minutes of Birth Compared to Type of Parturition

**Figure 3:**
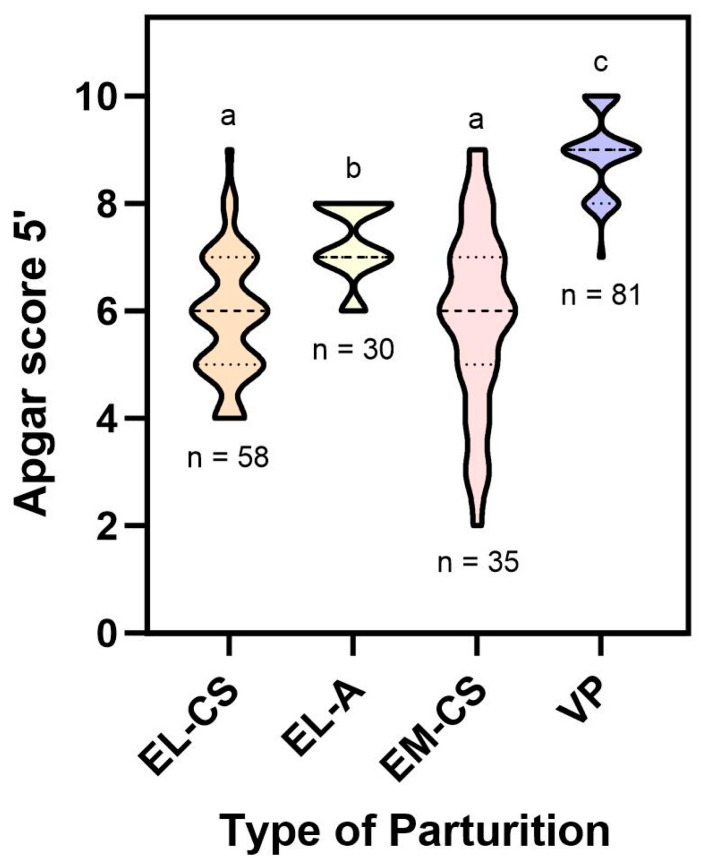
Apgar score measured in first 5 min after birth compared to the type of parturition. Different small letters above the violin plots indicate significant differences (*p* < 0.05) between Apgar score and the type of parturition.

The VP group exhibited the highest Apgar score within 5 min of birth (8.9 ± 0.7), followed by the EL-A (7.3 ± 0.7) group, which showed a statistically significant improvement when compared to EM-CS (5.9 ± 1.7) and EL-CS deliveries (5.9 ± 0.6) (*p* < 0.05). In EM-CS deliveries, the lowest scores were primarily associated with the puppy responsible for dystocia, while the scores in other puppies were considerably better. Conversely, nearly all puppies in the EL-CS group exhibited slightly lower Apgar scores (Figure 3). One hour after birth, there were no statistically significant differences in the Apgar scores between groups, and all puppies showed Apgar scores of nine or ten. 

Lower IgG and IgA concentrations in EL-CS did not have an impact on puppy survival (*p* > 0.05). The lower IgG and IgA concentrations in EL-CS had no effect on the survival of the puppies (*p* > 0.05). Seven puppies (0.09%) died in the VP group, four (0.07%) in the EL-CS group, three (0.08%) in the EM-CS group, and two (0.07%) in the EL-A group.

## 4. Discussion

The newborn’s adaptation to extrauterine life depends on the availability and quality of colostrum. The concentration of maternal IgG absorbed after birth varies considerably between pups and between litters, with 18% of newborns suffering from poor passive immune transfer [11]. One of the reasons for this IgG deficit could be the intake of low immune quality colostrum, which could be influenced by the type of parturition, as shown in this study where the EL-CS had significantly lower IgG and IgA concentrations when compared to all other groups. Since any Ig deficit could jeopardize the survival of the newborn, a suitable substitute that is capable of supporting the immune system and energy must be provided [25].

In this study, IgG and IgA concentrations were measured in the colostrum of bitches undergoing different types of parturition. The IgG concentration in colostrum varied between 0.8–43.7 g/L at birth, which is consistent with previous studies [10,26]. These differences in IgG concentration likely reflect the diversity and individual characteristics of each bitch, indicating the complexity of colostrum production and the influence of genetic, nutritional, and environmental factors [10,26]. However, according to our study, the type of parturition is also influential to this complexity. The EL-CS group had a significantly lower IgG concentration compared to all other groups, underlining the influence of the delivery method on colostrum quality. Furthermore, previously reported IgG absorption rates were influenced by the timing of colostrum administration, and the IgG concentrations in puppy serum were significantly higher 48 h after administration when colostrum was ingested at 0–4 h compared to 8–12 h or 16–24 h. According to Chastant-Maillard et al. [27], this is due to a rapid closure of the intestinal barrier, but a colostral IgG drop in the first hours after birth could also be the reason. 

The IgA concentration in colostrum ranged from 0.6–25.7 g/L at birth to 2.0–21.8 g/L four hours later. The mean IgA concentration at birth was 13.7 g/L ± 5.8, and decreased to 11.7 g/L ± 4.6 four hours after birth, However, the difference was not significant. The lower IgA concentration compared to IgG is consistent with previous studies which found that IgG is predominant and accounts for 60–75% of total Ig, while IgA accounts for 16–40% of colostral Ig, yet then becomes the most abundant Ig in milk [26,28]. Since Ig is transmitted both via the placenta and the colostrum, dogs have relatively high concentrations of IgA and IgG in the colostrum. The increased presence of IgA in milk is mainly due to plasma cells in the mammary tissue, which are part of the gut-associated lymphoid tissue (GALT). Lymphocytes from the GALT migrate into the mammary gland and connect the mother’s mucosal immune system, in particular, the immune system of the intestinal mucosa, with the secretory Ig repertoire of the mammary gland. Consequently, colostrum and milk contain specific antibodies against pathogens with which the intestine of the newborn come into contact. Secretory IgA in the intestinal lumen plays a crucial role in protecting the epithelial barrier through binding bacteria, toxins, and other large molecules, preventing their interaction with intestinal cells and limiting their ability to trigger a systemic immune response [29].

As with IgG, the EL-CS group had a significantly lower IgA concentration compared to the other groups. However, the administration of aglepristone 24 h before the induction of labor attenuated this decrease and brought IgG and IgA concentrations in line with those of the VP and EM-CS groups. The lower IgA concentrations in the EL-CS group could be explained by the fact that they are formed in the mammary gland, and the EL-CS group may not yet provide sufficient stimuli [4]. The higher IgG and IgA concentrations in the EL-A group, compared to the EL-CS group, are difficult to explain, and a more detailed investigation of the triggering of parturition is required. 

Cortisol is crucial for triggering parturition in sheep and goats [28], but elevated cortisol levels in dogs do not appear to be mandatory for triggering the birth cascade [14,30,31]. Progesterone (P4) plays a critical role in the maintenance of pregnancy in dogs, particularly in the induction of prepartum luteolysis and the initiation of parturition [25]. Prepartum luteolysis in pregnant bitches is characterized by a rapid decrease in P4 levels around day 60 of the corpus luteum (CL) lifespan. The use of P4 signaling interrupters such as aglepristone underscores the central role of P4 as a luteotropic factor which is critical for the maintenance of pregnancy in dogs [32]. Therefore, the administration of aglepristone shortly before parturition results in effective labor in 100% of bitches, and can support lactation when elective cesarean section is considered [33]. 

In the last weeks of gestation, IgG from the dam’s blood is taken up by the cells of the mammary gland by specific receptors, i.e., neonatal fragment constant receptor (FcRn), which leads to an accumulation in the breast tissue. As a result, IgG is released into the mammary gland, which typically reaches levels which are 3–4 times higher than in the maternal bloodstream [4,10].

Feto-maternal communication between the decidual cells originating from the maternal stroma and the fetal trophoblast has been shown to be a crucial element in the regulatory mechanisms that initiate parturition in dogs. This process is closely associated with an increase in utero-placental PGF2α production [14,34], potentially influencing the release of IgG concentrations in the colostrum. Therefore, the concentration of IgG and IgA could be higher in the EL-A group than in the EL-CS group. In an EL-CS group at the time of ovulation, the puppies are suddenly and forcibly removed, potentially disrupting natural hormonal rhythms. In contrast, if an EL-CS is combined with the administration of aglepristone (EL-A) or performed as an EM-CS, the hormonal patterns are similar to those of natural birth. EL-A triggers labor hormonally, while EM-CS takes place when labor has already started. As a result, puppies delivered with EL-A and EM-CS have Ig levels similar to puppies born vaginally, suggesting a better synchronization of Ig production and mammary gland development with birth.

As demonstrated in this study, neonatal viability was better in the EL-A group than in the EL-CS and EM-CS groups. The administration of aglepristone and the subsequent withdrawal of progesterone may have helped to prepare the puppies for birth and thus achieve better Apgar scores at birth.

Although the levels of IgA and IgG in the EL-A group were statistically comparable to those of the VP and EM-CS groups, they were slightly lower, which may be due to agleprisotne-treated dogs having weaker uterine contractions and lower PGF2α levels compared to normally active VP. Importantly, unlike normal prepartum luteolysis, agleprisotne treatment does not induce an increase in glucocorticoid receptors (GR/NR3C1) in the utero-placental unit [35]. 

Moreover, as seen in this study, puppy viability after birth was better in the EL-A group when compared to the EL-CS and EM-CS groups, where aglepristone administration and the withdrawal of progesterone could again contribute to puppies preparing for birth. 

Although lower IgG and IgA concentrations were observed in the EL-CS group, the well-being of the puppies remained unaffected. Their survival rate was equally high in all four groups. It is noteworthy that an IgG serum level of 2.3 g/L in puppies requires an average colostrum intake of 1.3 mL per 100 g body weight within the first 8 h of life, taking into account an absorption rate of 40% in the digestive tract, a hematocrit of 35%, and a colostral IgG level of 20 g/L [4,35]. In an earlier study, the neonatal mortality rate within the first 21 days after birth was 44% in puppies whose concentration was below the threshold and 4.9% in those that exceeded it [4]. Thus, considering the survival rate of puppies even in the EL-CS group (3/58 puppies; 5.2%), where IgG and IgA levels were lowest, it can be concluded that they reached sufficient levels to adequately protect themselves against infectious diseases. Local immunity may also have contributed to the survival of the puppies. IgA ingested before the closure of the intestinal barrier is rapidly excreted through the mucous membranes, especially through the respiratory and digestive tracts, where it plays a role in local immunity [4,26]. It is important to mention that the general immunity of the newborn puppies could also be achieved because the closure of the intestinal barrier does not terminate the immunological function of the colostrum. The immunoglobulins ingested with the milk, including IgG and IgA, which are deposited in the digestive lumen, play a role in local immunity within the digestive tract. They either intercept pathogens or help present antigens to white blood cells, thus contributing to immunity [4,36]. It is important to acknowledge a major limitation of our study, namely the fact that the composition of the birth groups was determined by randomization based on the animals brought to the clinic. This resulted in different breeds being represented in the different groups. It would be beneficial for future studies if the same breeds were deliberately represented in all groups. This approach would allow a more meaningful comparison of colostrum composition between the different types of births, providing a clearer understanding of the differences observed.

## 5. Conclusions

To summarize, the birth process is a complex interplay of hormones with a precise schedule, especially in dogs. While the prepartum progesterone drop is helpful, other hormones have limited clinical utility in predicting the onset of labor, especially in cases where a cesarean section is required prior to birth. The results of this study suggest that the withdrawal of progesterone prior to labor may contribute to higher IgG and IgA concentrations in the mammary glands of bitches, and that the administration of aglepristone prior to an elective cesarean section could improve the quality of birth and also the viability of puppies immediately after birth. Further research is essential to unravel the nuances of these intricate processes and their potential implications for clinical use.

## Figures and Tables

**Table 1:** Number and proportion (%) of different breeds included in the study and their distribution regarding the type of delivery.

Breed Size	Breed	Number of Parturitions (%)	Type of Parturition (Number) *	Number of Puppies (%)
Small breeds (<10 kg)	Cavalier King Charles Spaniel	1	EL-A (1)	4
	Yorkshire Terrier	4	VP (1), EL-CS (2), EM-CS (1)	17
	Miniature poodle	2	VP (1), EM-CS (1)	8
	Boston Terrier	5	VP (3), EL-CS (1), EM-CS (1)	21
	Total small breeds	12 (30.0)		50 (24.5)
Medium breeds (10–25 kg)	English Bulldog	3	EL-CS (3)	11
	French Bulldog	3	EL-CS (3)	10
	Miniature bull terrier	3	VP (2), EM-CS (1)	16
	Beagle	1	EL-A (1)	5
	Dachshund	1	VP (1)	6
	Whippet	2	VP (1), EL-A (1)	11
	Pembroke Welsh Corgi	3	VP (2), EM-CS (1)	10
	Total medium breeds	16 (40.0)		69 (33.8)
Large and giant breeds (>25 kg)	Greater Swiss Mountain Dog	3	EL-CS (1), EM-CS (1), EL-A (1)	21
	Karst Shepherd	2	EL-CS (1), EM-CS (1)	10
	Labrador Retriever	2	EM-CS (1), EL-A (1)	13
	German Shepherd	1	VP (1)	9
	Golden Retriever	1	EL-A (1)	8
	Dogo Argentino	1	EL-A (1)	7
	Great dane	2	VP (1), EL-A (1)	17
	Total large and giant breeds	12 (30.0)		85 (41.7)

* the number of bitches of a particular breed that have given birth with a particular type of delivery.

**Table 2:** Number and proportion (%) of puppies according to the type of parturition, sex, and survival of the newborns.

		Number of Puppies	Proportion of Puppies [%]
Type of parturition	VP	81	39.7
	EL-CS	58	28.5
	EM-CS	30	17.1
	EL-A	35	14.7
Sex of the puppies	Females	106	51.9
	Males	98	48.1
Survival	Born alive	204	
	Died after discharge	16	7.8

**Table 3:** Number of puppies in each group according to breed size.

Breed Size	Number of Puppies			
	VP	EL-CS	EM-CS	EL-A
Small breeds	22	17	7	4
Medium breeds	38	21	4	6
Large and giant breeds	21	20	19	25
Total	81	58	30	35

## Data Availability

Data are available from the authors upon request.
